# Self-Sensing CFRP Fabric for Structural Strengthening and Damage Detection of Reinforced Concrete Structures

**DOI:** 10.3390/s18124137

**Published:** 2018-11-26

**Authors:** Qian Feng, Jinping Ou

**Affiliations:** 1 Institute of Engineering Mechanics, China Earthquake Administration. Key Laboratory of Earthquake Engineering and Engineering Vibration, China Earthquake Administration, Harbin 150080, China; oujinping@hit.edu.cn; 2 Hubei Key Laboratory of Earthquake Early Warning, Institute of Seismology, China Earthquake Administration, Wuhan 430071, China; 3 Wuhan Institute of Earthquake Engineering Co. Ltd., Wuhan 430071, China; 4 Key Laboratory of Structures Dynamic Behavior and Control of the Ministry of Education, Harbin Institute of Technology, Harbin 150090, China

**Keywords:** piezoceramic transducers, self-sensing carbon fiber reinforced polymer (CFRP) fabric, reinforced concrete beams, damage detection

## Abstract

This paper presents a concept of a Self-sensing Carbon Fiber Reinforced Polymer (SCFRP) system, which integrates the piezoceramic transducers with the common concrete strengthening materials, CFRP fabric. This integration provides the SCFRP fabric with the ability to monitor the structural health condition when the SCFRP fabric is applied on reinforced concrete structures. In order to validate the feasibility of this system, several three-point bending beam (3PBB) specimens were fabricated and tested before and after the specimens were reinforced with the proposed SCFRP fabric. In addition, the specimens with the low (C25) and high (C40) concrete grades were also experimentally investigated to evaluate the reinforced effectiveness of the SCFRP fabric. Finally, the experimental results demonstrate that the proposed SCFRP fabric can significantly improve the bearing capacity of the concrete structures, and provided the reinforced concrete structures with an ability of self-sensing their health condition.

## 1. Introduction

In recent years, various Fiber Reinforced Polymers (FRPs) have received much attention in the broad engineering community [1,2,3,4]. Among FRPs, the use of Carbon Fiber Reinforced Polymers (CFRPs) as strengthening materials has attracted the interest of many researchers due to their outstanding advantages, including strong fatigue and corrosion resistance, light weight, and high strength [5,6,7,8]. These advantages have helped CFRPs find wide application in the fields of aerospace, sport and automobile industries. In civil engineering, the CFRPs are commonly used in steel and concrete reinforced structures, for example, for structural retrofitting and repair [9,10,11,12]. 

The use of CFRP offers civil engineers an attractive solution to structural strengthening. Compared to other conventional strengthening techniques, CFRP has the advantages of light weight, high mechanical strength, high resistance to corrosion, high durability, convenient in-situ application and nearly no influence on the structure [13,14,15]. Extensive previous studies have focused on the mechanical properties of CFRP, including the effect on global stiffness and strength, bond force transfer and development, and fatigue performance of CFRP-strengthened structures. For example, El-Ghandour [16] investigated the efficiencies of flexure and shear strengthening of the CFRP materials on concrete beams. Spadea et al. [6] studied the structural behavior of reinforced concrete beams strengthened with externally bonded CFRP sheets, and the experimental data indicated that bonding a CFRP plate on the tension force of a concrete beam can lead to significant degradation in the structural response of the plated beam. Gherdaoui et al. [17] tested the punching behavior of reinforced concrete slabs strengthened with CFRP, and the results illustrated that CFRP strengthening represents a good performance in reducing the deflection of structures. Dai et al. [18] proposed a new analytical method for defining the nonlinear bond stress-slip models of FRP sheet-concrete interfaces through a series of pullout tests. In their method, the strain distributions in FRP as well as the local bond stresses can be simply derived without attaching any gauges on the FRP sheets. Rousan and Issa [19] presented an experimental and analytical study involving the static and accelerated fatigue testing performance of reinforced concrete beams externally strengthened with different number and configuration of CFRP sheets. In addition, these authors recommended fatigue design considerations for calculating the reduction in the stiffness and ultimate load capacity due to fatigue loading. In summary, CFRP materials have been successfully applied to various practical projects. For example, Miller et al. [5] took advantage of CFRP plates to strengthen an existing structurally deficient steel bridge. Borri et al. [20] demonstrated a method for flexural reinforcement of existing old wood elements under bending loads. 

However, for the reinforced concrete structures, how to evaluate the strengthening quality and the subsequent working status of the CFRP-strengthened structures still faces lots of challenges during their service period. In recent years, increasing interests has developed in the adoption of smart sensing technologies for instrumentation within a variety of structural systems. For the intermediate crack debonding in FRP strengthened reinforced concrete structures, Ricardo et al. [21] developed a multi-objective model updating approach combined with the electro-mechanical impedance technique to identify the debonding damage in reinforced concrete flexural beams strengthened with FRP. Seung et al. [22] proposed a reference-free nondestructive testing (NDT) to continuously inspect the bonding condition between carbon fiber-reinforced polymer (CFRP) and host reinforced concrete (RC) structures, and the time reversal acoustics (TRA) technique was applied and helpd in debonding detection without relying on previously-obtained baseline data. 

Structural health monitoring (SHM) [23,24,25], which employs integrated transducers to monitor structural status in real time [26,27,28,29] so that early warnings can be issued to prevent catastrophic events using pre-developed algorithms [30,31,32,33], has received much attention [34,35,36,37]. As an enabling device in the field of SHM, lead zirconate titanate (PZT), a type of piezoceramic material, has been widely used due to its low cost, fast response, availability in different shapes, and capabilities as both an actuators and a sensor [38,39,40,41,42,43]. By taking advantage of these features, PZT transducers have been studied for a promising application to civil engineering structures by many researchers, such as Song et al. [44], Yang et al. [45], Park et al. [46], Wang et al. [47], Feng et al. [48,49]. In these applications, the PZT transducers were embedded in [50,51,52,53] or bonded on [54,55,56,57] the host structure, and the health condition of the structures can be achieved by analyzing the stress wave propagation and attenuation when compared to the signatures under the undamaged condition [58,59,60]. Unfortunately, few literatures have reported the PZT-based health monitoring of strengthened concrete structures during its service period, to the authors’ best knowledge. 

In this study, a novel Self-sensing Carbon Fiber Reinforced Polymer (SCFRP) fabric is developed to integrate the damage detection capability of PZT transducers with the strengthening ability of CFRP fabric. The first part of this paper introduces the detection principle and the specification of the SCFRP fabric. Several pairs of piezoceramic transducers, which had been integrated to the reinforced FRP materials, are surface-bonded on the host structure, and thus a piezoceramic-based active sensing system can be built to monitor the health condition of the FRP-reinforced concrete. In the study, several concrete beams with different strengths and reinforced with SCFRP fabric were prepared as the test specimen. A pair of piezoceramic transducers is bonded on the surface of the to-be-reinforced structure in the desired location before the CFRP fabric reinforcement. Then, three-point bending tests are applied on these specimens, and the stress waves propagated along the specimen are acquired and analyzed during the loading procedure. The wavelet packet energy algorithm is employed to analyze the received signal to discuss and evaluate the health condition of the reinforced structures. 

## 2. SCFRP-Based Health Monitoring Technique

### 2.1. Concept of SCFRP and Its Applications

The novel SCFRP fabric is fabricated by integrating waterproofed PZT patches with the carbon fiber polymer fabric, taking advantage of the CFRP’s high strength and piezoceramic transducers’ active sensing capacity. Therefore, the cracks or debonding damage in the strengthened concrete structures can be detected with the help of novel SCFRP fabric by analyzing the propagation and attenuation characteristics of the stress waves between the pair of piezoceramic transducers located at desired location in the structures. The integrated PZT patches of the SCFRP fabric can be connected to the data acquisition device via BNC cables for data acquisition. 

**Figure 1:**
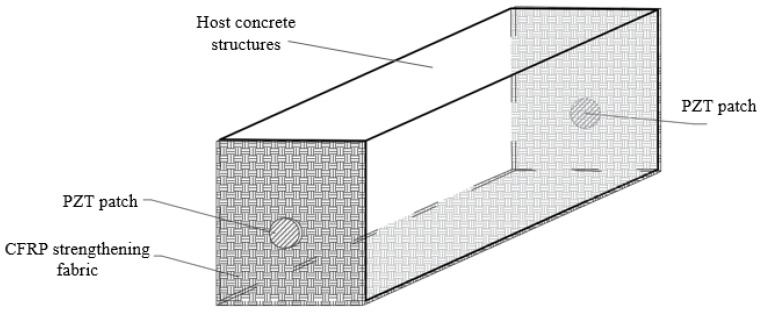
The schematic diagram of SCFRP fabric for strengthening the structures.

**Figure 2:**
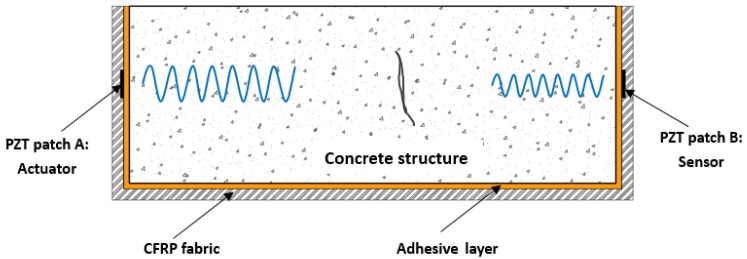
The damage detection principle of the SCFRP fabric.

Similar to the common CFRP strengthening material, the proposed SCFRP fabric integrated with the piezoceramic patches can also be used to strengthen the concrete structures by surface bonding them onto the host structures, as shown in Figure 1. The integrated PZT patches that face each other were installed at the desired locations during the strengthening operation. Due to the special characteristic of piezoceramic material which can function as an actuator and a sensor, one PZT patch as the actuator driven by the laptop with a desired voltage excitation can submit a stress wave, which then propagate through the strengthened concrete structure. Then, the attenuation of the stress wave was finally received by the other PZT patch, which was regarded as the sensor located at the opposite location of the actuator. Upon the excitation, the generated stress wave propagated through the concrete structure, interrogating the integrity of the host structure, and thus providing information on the actual health condition of the strengthened structures. Thus in the practical applications, the integrity and damage condition of the SCFRP-based strengthening concrete structure can be monitored and evaluated by analyzing the received attenuation signatures of the sensors installed on the SCFRP fabric, as shown in Figure 2. 

### 2.2. Wavelet Packet Energy-Based Monitoring Index

In the practical applications, the proposed SCFRP fabric was employed to strengthen concrete structures following the conventional strengthening procedure. Then, a dataset of the stress wave signatures acquired from the SCFRP fabric of the strengthened structure was collected as the baseline when the strengthening work was finished. Thus, the health condition of the strengthened concrete structure in its service period can be monitored in real time by comparing the acquired wave response with the baseline. 

To quantitatively estimate the differences between the stress wave responses, the wavelet packet energy technique was employed here to build a monitoring index to evaluate the health condition of the structures [61,62,63,64]. As one of the effective techniques to analyze the transmitted signal between two PZTs, wavelet packet based approach has been widely used in recent years [65,66]. A waveform that has an average value of zero in a limited duration can be called a wavelet. In wavelet analysis, a signal can be divided into two parts, low frequency and high frequency, which are approximation and detail, respectively. The approximation itself can be divided into a second level approximation and detail. The detail can also be spilt the same way as approximation in wavelet packet analysis. The outstanding feature of wavelet packet analysis is that it makes inspection of relatively narrow frequency bands during a relatively short time window possible. In this paper, wavelet packet-based energy analysis is developed to define the energy of the sensor under different test condition compared to the baseline. 

The sensor signal, S, is decomposed by *n*-level wavelet packet decomposition into 2*n* signal sets {*X*_1_, *X*_2_, ⋯, $X_{2^{n}}$}. In each signal set *X_j_*, where *j* is the frequency band (*j* = 1, 2, ⋯, 2*^n^*). In this study, *n* = 5. *X_j_* can be further expressed as:

(1) $$X_{j}=[x_{j,1},\;x_{j,2},\cdots ,\;x_{j,m}]$$

where *m* is the number of data samples in each signal set. Thus, the energy of the signal set can be calculated as:

(2) $$E_{j}\;=\;\sum_{k=1}^{k=m}x_{j,k}^{2}\;$$

Therefore, the total energy of the signal can be calculated by the summation of all the signal sets. The total energy of the signal can be expressed as:

(3) $$E=\sum_{j=1}^{i=2^{n}}E_{j}\;$$

Therefore, the received signal can be characterized by using the energy value based on the wavelet packet energy analysis. In addition, the energy values calculated from the received signals under different working condition for the SCFRP-based strengthened concrete structure can also be easily compared. 

## 3. Experimental Setup and Procedures

### 3.1. Experimental Specimens

To validate the effectiveness of the SCFRP fabric technology in the area of the structural health monitoring and strengthening, several concrete beam specimens were first prepared in the laboratory, then a three-point bending test was conducted for the concrete specimens with and without the SCFRP fabric. In addition, two kinds of concrete specimens with different concrete grade (C25 and C40) were also prepared to investigate the influence of concrete strengthening. 

**Figure 3:**
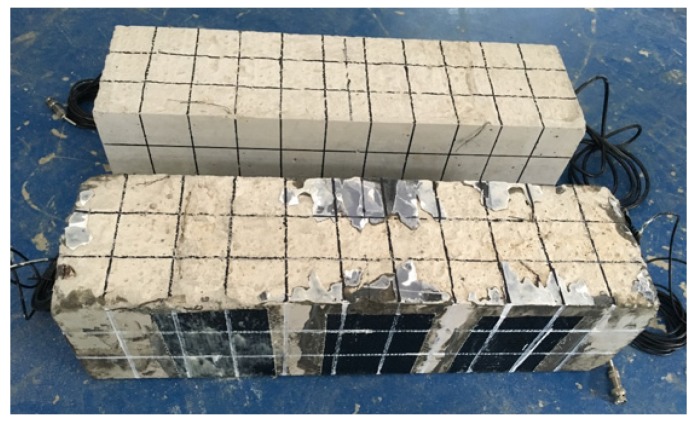
Specimens of concrete beams strengthened with and without CFRP fabric.

As shown in Figure 3, the geometric dimensioning of the concrete beam specimen is 150 × 150 × 550 mm. Four different specimens were involved in the experimental investigation: *specimen 1*: a C25 concrete beam without SCFRP fabric strengthening; *specimen 2*: a C25 concrete beam strengthened with SCFRP fabric; *specimen 3*: a C40 concrete beam without SCFRP fabric strengthening; and *specimen 4*: a C40 concrete beam strengthened with SCFRP fabric. 

In this study, three-point bending tests were carried out by a universal hydraulic testing machine (100T) to verify the feasibility of the proposed SCFRP fabric. For the convenient observation of the possible crack development during the test, the surface of the specimens was meshed, as shown in Figure 3, as squares with a size of 50 mm by 50 mm. 

For the specimen beam with strengthening of SCFRP fabric, the SCFRP fabric with the piezoceramic transducers was surface bonded on the concrete specimen with the structural adhesive, as shown in Figure 2. In this kind of specimen, the two PZT patches were installed on the beam with the face-to-face configuration, as shown in Figure 1. The material characteristics of the concrete and CFRP fabric were test following the related standard specification, and the test results are presented in Table 1, in which, the material parameters of the structural adhesive and PZT patches are also included. Thus, the crack and damage occurred along the length direction of the beam can be detected using the proposed technique. 

### 3.2. Experimental Setup and Test Procedure

**Figure 4:**
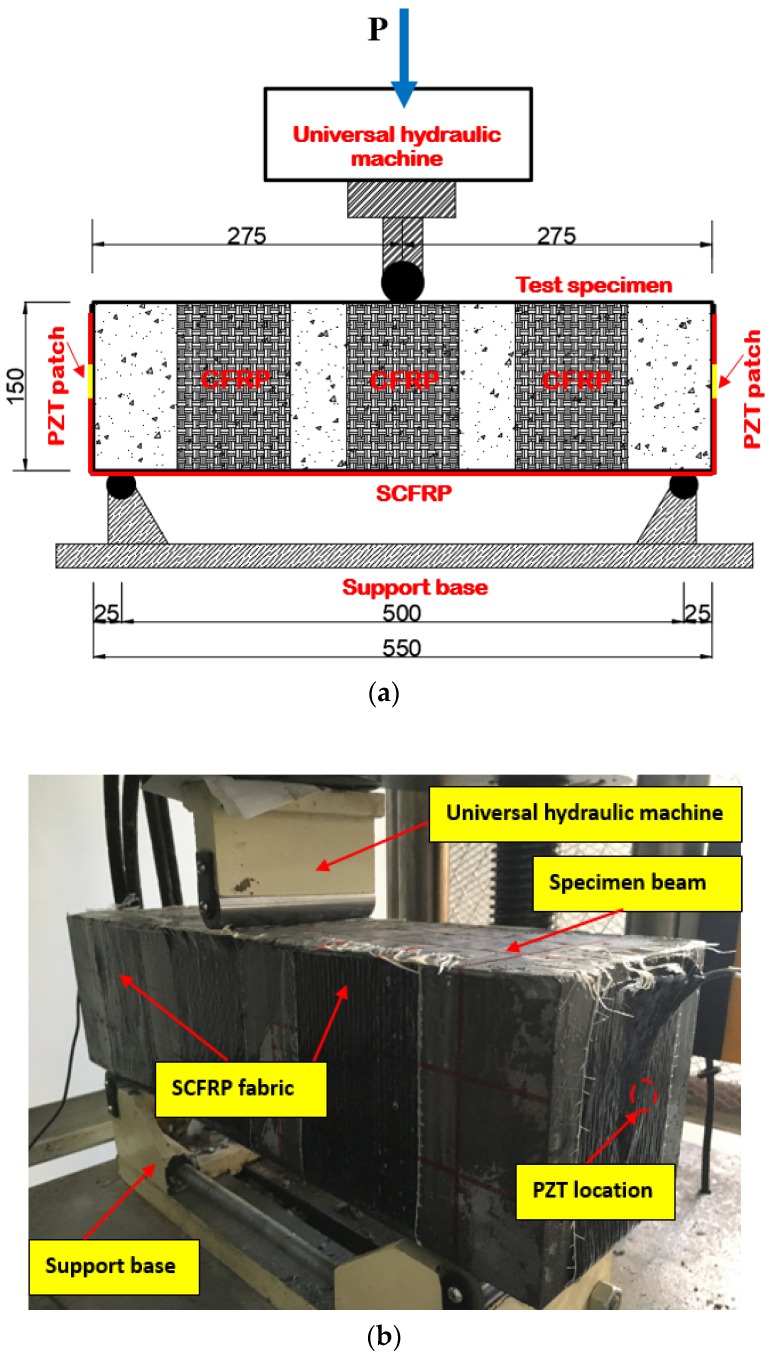
Experimental setup: (**a**) detail loading setup and geometric dimension (mm), (**b**) the actual test setup.

In the study, a 100T capacity universal hydraulic testing machine was employed in the experiment to apply an axial compressive load on the specimen with the force-control. As shown in Figure 4, the test specimen was placed on a steel support base, then the vertical load was applied onto the central position of the specimen by the hydraulic machine. The detail geometric dimensioning of the steel base and load applied location was also presented in the Figure. Thus, a three-point bending test can be conducted on the SCFRP fabric strengthened concrete specimen beam. 

**Figure 5:**
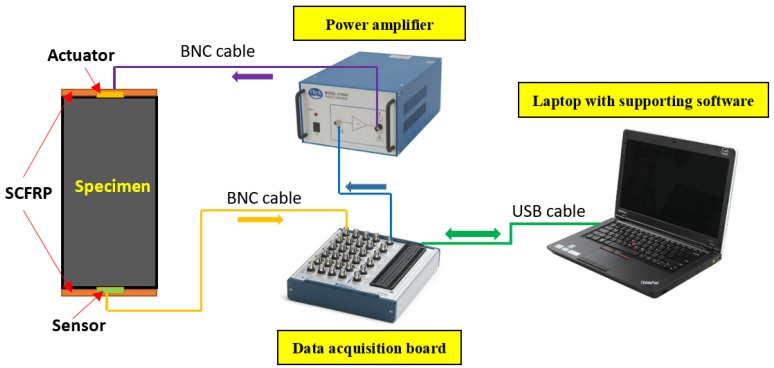
Procedure of the SCFRP system.

Following with strengthening operation of the SCFRP fabric, the PZT transducers were also surface bonded onto the SCFRP strengthened concrete specimen beams. Then, the PZT patches were connected with the data acquisition board (NI-6363), which was controlled by a laptop installed with the supporting software, as shown in Figure 5. For each test, a load was applied on the specimen with the force-control at a desired rate. Once reaching the target load value, a 240-s holding period was used to complete the procedure of data acquisition of the SCFRP fabric. Under different load conditions, a swept voltage excitation signal was first generated from the data acquisition board, and then transmit to the PZT actuator. Upon the excitation, a stress wave was generated from the actuator and then propagated through the concrete specimen. The other PZT patch as the sensor detected the stress wave response and converted it to an electrical signal, which was acquired by the computer via A/D interface of the NI data acquisition card. 

For each test, a swept sine signal with the frequency band of 100 Hz to 400 kHz was employed as the excitation signal. The sampling frequency of the data acquisition is 2 MHz with a period of 0.5 s, which was sufficient to prevent or minimize the aliasing effects.

## 4. Experimental Results and Analyses

### 4.1. Experimental Results for Four Different Conditions

(a) *Specimen 1*: C25 concrete without SCFRP fabric strengthening 

**Figure 6:**
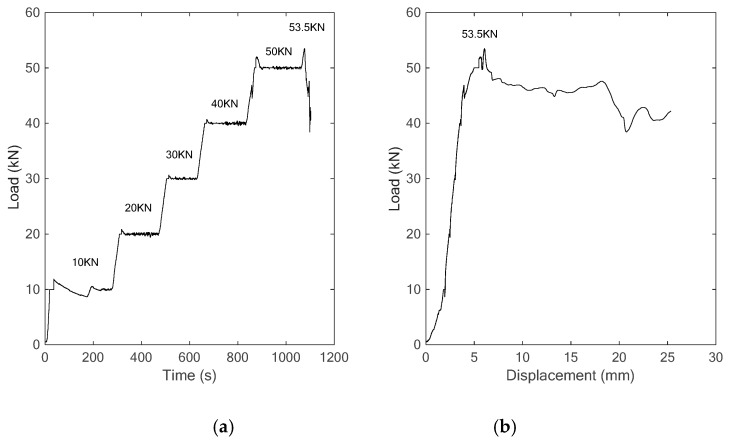
The curves of (**a**) time vs. load, (**b**) displacement vs. load.

**Figure 7:**
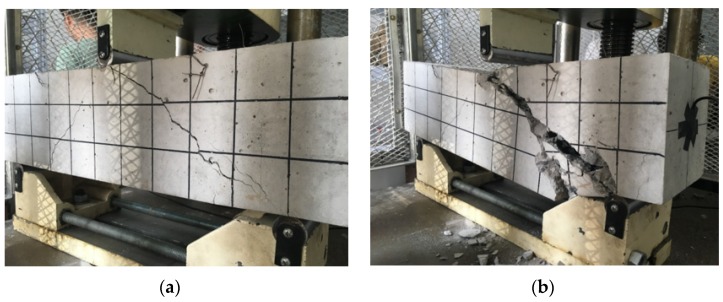
Failures of the specimen (**a**) cracks at load of 20 kN, (**b**) collapse at load of 53.5 kN.

Following the procedure described above, the specimen with the concrete strength of C25 was first employed as the object for the three-point bonding test. In the experiment, the applied vertical load increased gradually with an interval of 10 kN, and finally reached to the maximum value of 53.5 kN. The load-time history and load-displacement relationship are shown in Figure 6. In addition, two photos of the occurrence and development of the crack in the specimen at different load levels are also shown in Figure 7. 

Similar to Figure 1, to build a baseline dataset of the stress wave propagation, two PZT transducers were surface bonded on the specimen. A swept sine signal with the frequency band of 100 Hz to 400 kHz and a time duration of 0.5 s was applied on the PZT actuator, and the corresponding wave response was collected by the PZT sensor.

**Figure 8:**
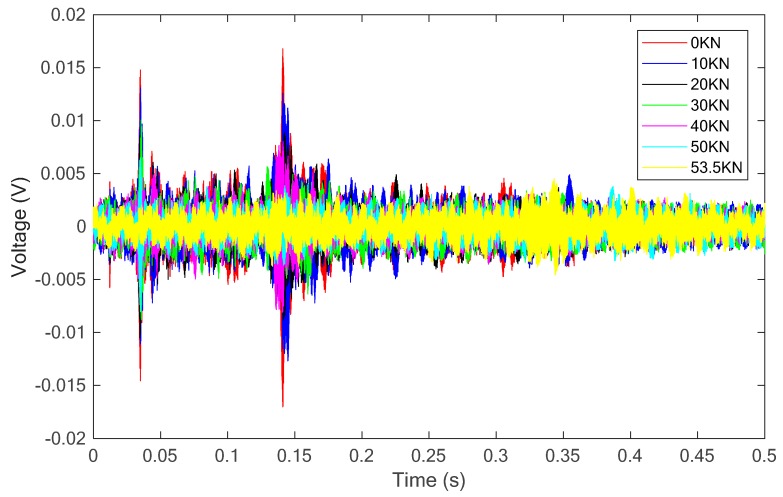
The received signal of the sensor for the specimen without SCFRP fabric strengthening.

Figure 8 presents the received signal of the sensor of the test specimen under this condition. From the figure, it is clear that the magnitude of the received signal decreases with the increase of the applied load on the specimen. 

**Figure 9:**
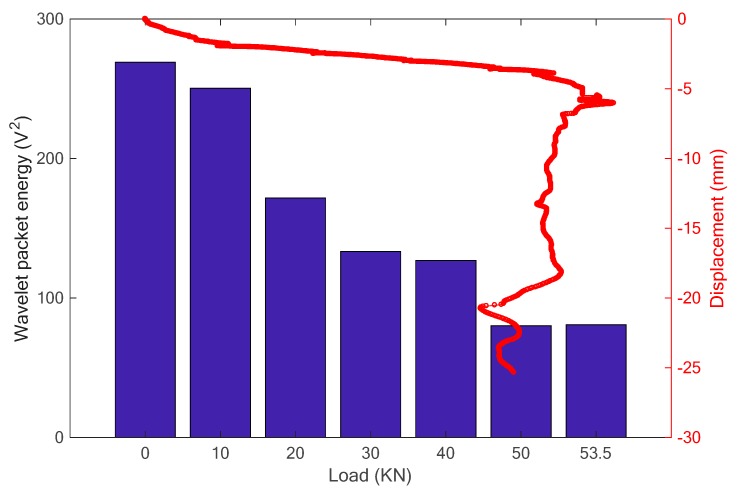
The results of wavelet packet energy.

Then, the wavelet packet energy of each received signal was computed and is plotted, as shown in Figure 9. In the figure, the relationship of displacement-load is also presented to more clearly analyze the changing trend. It is observed that the computed wavelet packet energy of the received signals decreases with the increase of the load, which is consisted with the development of the specimen displacement. 

In the study, it is predicted that the decrease of the wavelet packet energy was caused by the occurrence and development of the crack, which significantly influences on the stress wave propagation between the pair of piezoceramic patches. With the increase of the applied load, the displacement increases and cracks start to appear and widen, which attenuates the stress wave propagation from the PZT actuator to the PZT sensor. For example, the occurrence of an initial crack at the load of 20 kN (as shown in Figure 7a) directly induced a sudden drop for the received stress wave energy presented in Figure 9, when compared to the undamaged condition. Then, as the crack develops, the energy of the stress wave decreases gradually until the specimen collapses. Therefore, it is validated that the piezoceramic-based active sensing technique has the ability to monitor the health condition and detect the structural damage of the concrete structures. 

(b) *Specimen 2*: C25 concrete strengthened with SCFRP fabric

Then, the specimen of the concrete beam, which was fabricated with the C25 concrete and strengthened with the proposed SCFRP fabric, was investigated using the three-point bending test. 

**Figure 10:**
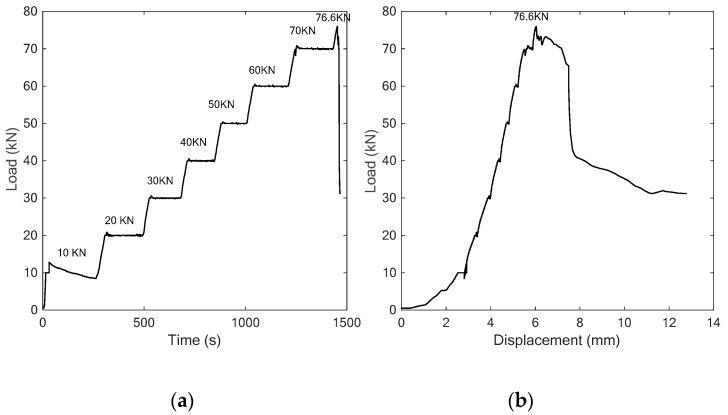
The curves of (**a**) time vs. load, (**b**) displacement vs. load.

**Figure 11:**
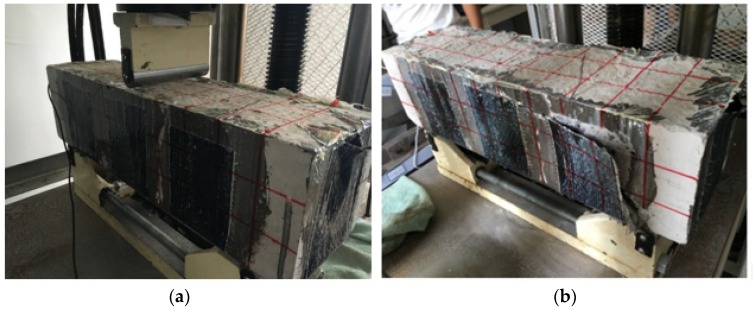
Test procedure (**a**) initial condition (**b**) collapse at load of 76.6 kN.

Like the test procedure described above, a vertical load with an interval of 10 kN was applied to the specimen and finally the maximum load value of 76.6 kN was reached, as shown in Figure 10, which, in addition, shows the relationship between the load and the displacement. Different from the failure mode of the concrete specimen in the previous section, the SCFRP strengthening of the concrete specimen improves the ultimate bearing capacity of the test structure from 53.5 kN to 76.6 kN, and induces a very different failure mode during the test, as shown in Figure 11. For the concrete specimen without the strengthening, the compressive shear crack finally induces the failure. However, for this specimen, the collapse of the SCFRP-strengthened concrete specimen was observed by the debonding between the U-shaped CFRP strengthening fabric and the beam. 

For the specimen of C25 strengthened with the SCFRP fabric, with the help of structural adhesive layer, the SCFRP fabric and the concrete specimen work together to bear the outside applied load, thus the ultimate bearing capability of the strengthened concrete specimen is significantly improved. However, due to its low strength of the specimen concrete, the concrete protective cover firstly reach to its maximum bearing capability when compared with the CFRP fabric and adhesive layer, and then induce the debonding of the SCFRP fabric and finally induce the collapse of the whole structure, as shown in Figure 11b. 

**Figure 12:**
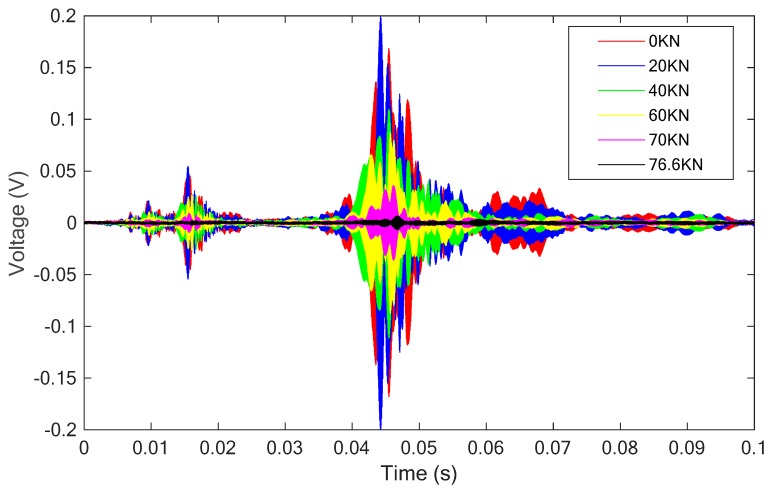
The received signals of the sensor for the specimen with the SCFRP strengthening.

For this specimen, the SCFRP fabric integrated with a pair of PZT-based transducers was surface bonded on it. With the same excitation as described in the previous sections, the received stress wave signals were recorded and are plotted in Figure 12. 

**Figure 13:**
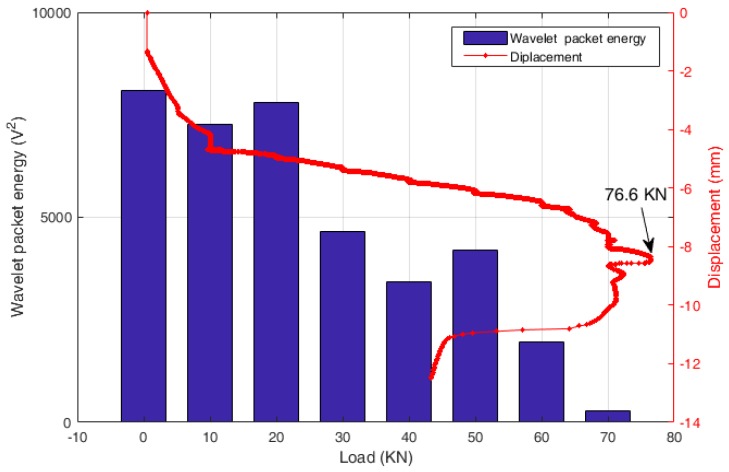
The result of wavelet packet energy.

In addition, the wavelet packet energy of the received signals were also computed, as shown in Figure 13. With the increase of the applied load, a crack occurred and widen during the test, and directly influence the stress wave propagation and attenuation, which can be represented by the change trend of the calculated wavelet packet energy values, as shown in Figure 13. From the Figure 12 and Figure 13, more attenuation can be observed for the received stress wave signal with the increase of the applied load when compared with the initial condition. 

The changing trend of the wavelet packet energy presented in the figure clearly demonstrates the whole process of the specimen failure. For example, during the experiment, with a big sound, a significant crack was observed on the surface of the specimen when the applied axial load was increased from 20 kN to 30 kN. This abnormality can also be clearly presented in the figure with a suddenly drop for the wavelet packet energy. Then, with the load increased from 50 kN to 60 kN, a local debonding of the SCFRP fabric occurred at the outermost U-shape fabric. Similarity, this damage induces more serious attenuation for the stress wave propagation and a significantly drop was observed for the wavelet packet energy in the figure. Finally, the specimen was complete failure due to the debonding between the U-shaped CFRP strengthening fabric and the beam, which induces a minimum for the energy value. Thus, the working status and health condition of the SCFRP-strengthened concrete structures can be evaluated in some degree by observing the variation of the wavelet packet energy of the propagated stress wave. 

Therefore, the proposed self-sensing CFRP fabric integrated piezoceramic transducers not only strengthen the concrete structure to improve its bearing capability similar to the common CFRP material, but also monitor the health condition of the strengthened concrete structure at the same time. 

(c) *Specimen 3*: C40 concrete without SCFRP fabric strengthening 

**Figure 14:**
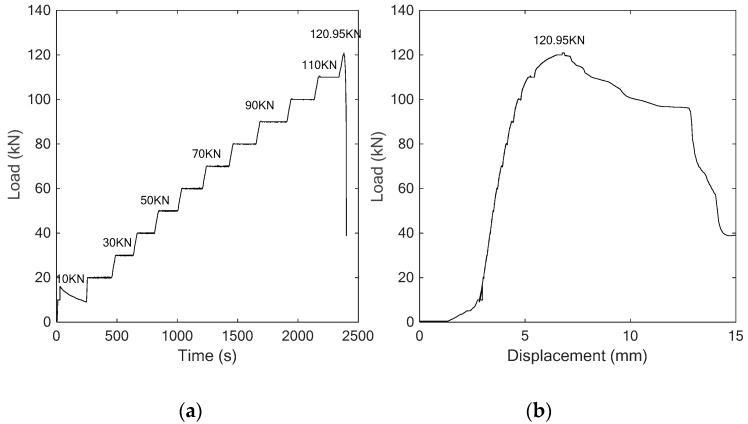
The curves of (**a**) Time vs. load, (**b**) Displacement vs. Load.

**Figure 15:**
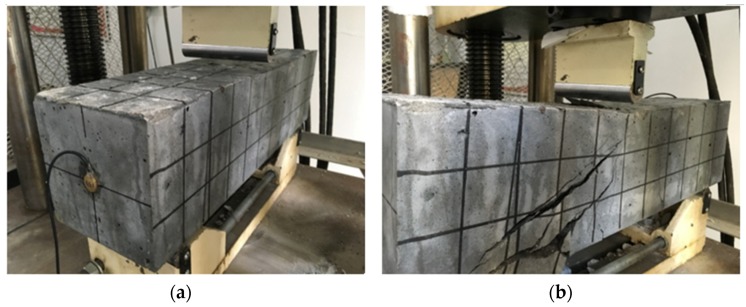
Test procedure (**a**) initial condition (**b**) collapse at load of 120.95 kN.

To investigate the performance of the proposed SCFRP fabric for the concrete structures with different concrete strength, specimens were also fabricated with C40 concrete and tested in the study. At first, this specimen without strengthening was subjected to the vertical load following the procedure of three-point bending test. The applied load history and the load-displacement relationship are presented in Figure 14. Benefit from the high-strength of the concrete, the maximum of the loading capability of the specimen reached to 120.95 kN, a much higher value than the previous two specimens. Figure 15 clearly reveals the failure mode of the specimen, which finally failed because of the compressive shear crack. 

**Figure 16:**
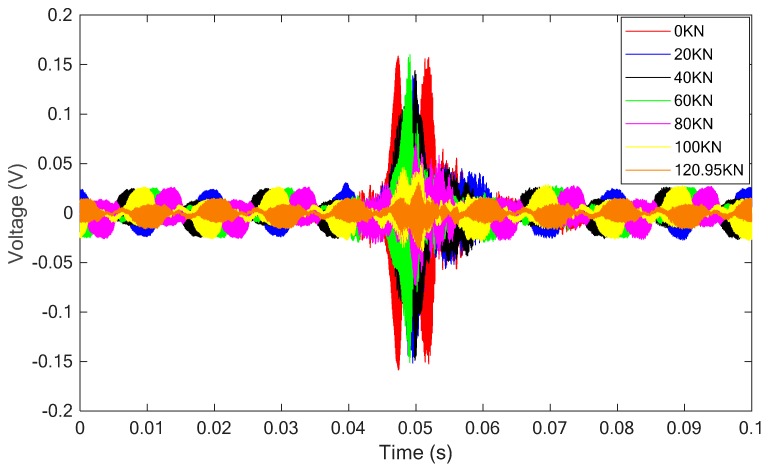
The received signal of the sensor for the specimen with the SCFRP strengthening.

**Figure 17:**
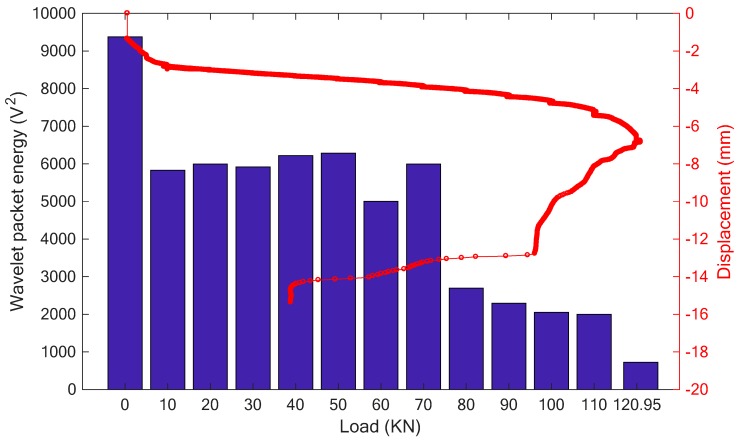
The result of wavelet packet energy.

Figure 16 and Figure 17 respectively present the received stress wave signals of the sensor and the computed wavelet packet energy value. Similar to the above analyses, the magnitude of the received signals decreases with the increase of the applied load. Furthermore, a good consistence was also observed between the change trend of the wavelet packet energy and the displacement. 

For this kind of specimen, the stronger bearing capability due to the higher concrete strength can also be represented in Figure 17. During the test, the first visible crack of the specimen was observed at the load of 80 kN, which can also be presented in Figure 17, in which, a significant drop occurred at this load stage. Then, as the crack develops, more attenuation for the stress wave can be observed as shown in Figure 16, which finally induces the decrease of the received energy of the sensor. Therefore, a similar conclusion can be verified that the piezoceramic-based active sensing has the ability to monitor the health condition of the concrete structures. 

(d) *Specimen 4*: C40 concrete strengthened with SCFRP fabric

**Figure 18:**
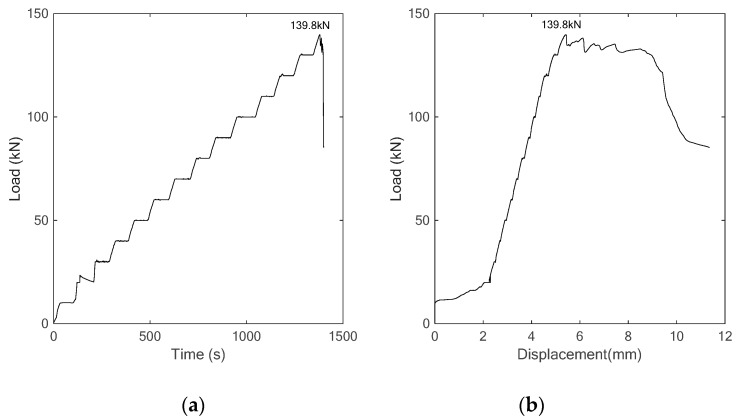
The curves of (**a**) time vs. load, (**b**) displacement vs. load.

**Figure 19:**
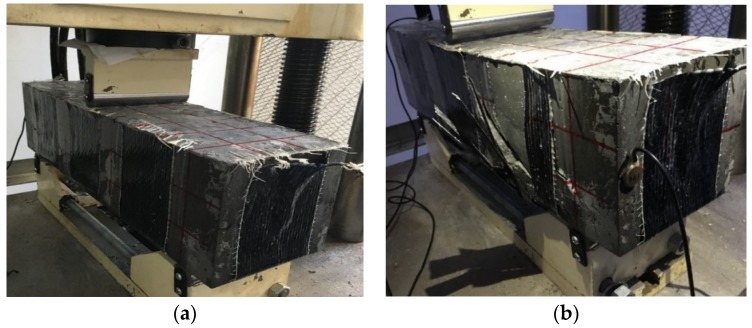
Test procedure (**a**) initial condition (**b**) collapse at load of 139.8 kN.

**Figure 20:**
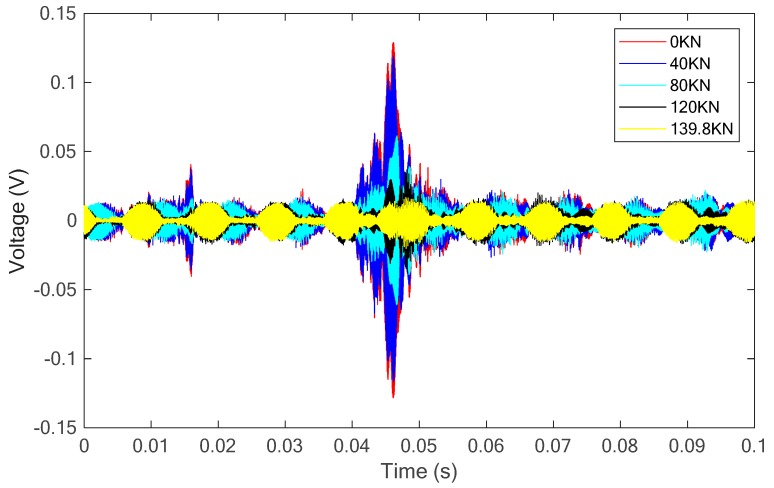
The received signals of the sensor for the specimen with the SCFRP strengthening.

**Figure 21:**
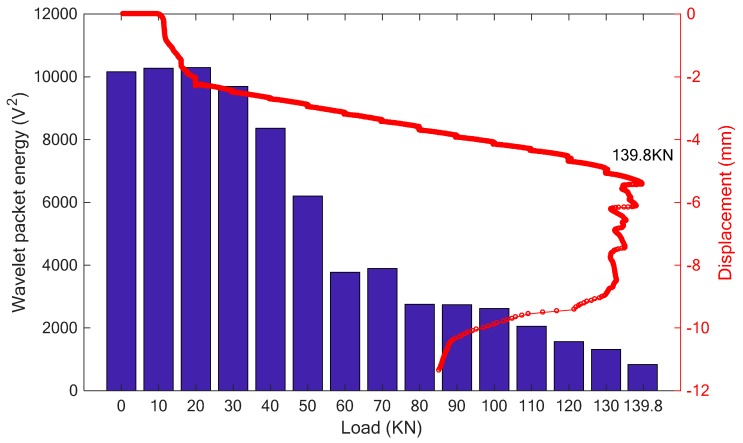
The result of wavelet packet energy.

Finally, the C40 concrete specimen strengthened with the proposed SCFRP fabric was tested and investigated. Figure 18 presents the time history of load and the load-displacement relationship during the test. From the figure, it is observed that the loading capability of specimen is 139.8 kN, which represents a significant improvement compared to the three previous specimens. In addition, Figure 19 shows two photos of the specimen under the initial condition and the final failed condition at the load of 139.8 kN. Upon the same swept sine excitation, the received stress wave signals of the sensor were collected and are plotted, as shown in Figure 20. At the same time, the wavelet packet energy of each received signal was also computed and is presented in Figure 21. 

In comparison to the specimen with concrete strength of C40 without strengthening, the bearing capacity of SCFRP fabric strengthened specimen increases from 120.95 kN to 139.8 kN, an improvement of 15.6%, which indicates that SCFRP strengthening fabric plays an important role in enhancing the bearing capacity of high-strength concrete structures. On the other hand, owing to the benefit of high-strength concrete, the bearing capacity of this kind of specimen also increased from 76.6 kN to 139.8 kN compared to the SCFRP strengthened specimen with C25 concrete. 

However, it should be noted that the failure modes of these two SCFRP strengthened specimen beams are not exact the same. From Figure 11 and Figure 19, it can be seen that the specimen beam with C25 concrete collapsed due to the debonding between the fiber reinforcement and the specimen body, while the specimen with C40 concrete failed because the carbon fiber reinforcement reached its ultimate bearing capacity. 

On the other hand, regardless of the high-strength or low-strength for the concrete, the SCFRP fabric integrated with the PZT transducer has the ability to monitor the health status of the concrete structures. In the experiment, a tiny crack was firstly observed at the load of 40 kN, and it was then further developed with the increase of the applied load until the SCFRP fabric reached to its maximum bearing capability, which will eventually result in the collapse of the entire specimen. From the Figure 21, a good consistence can be observed, in which, with the increase of loading and the development of cracks, the received energy of the stress waves gradually attenuates and represent the damage to the structure. 

**Figure 22:**
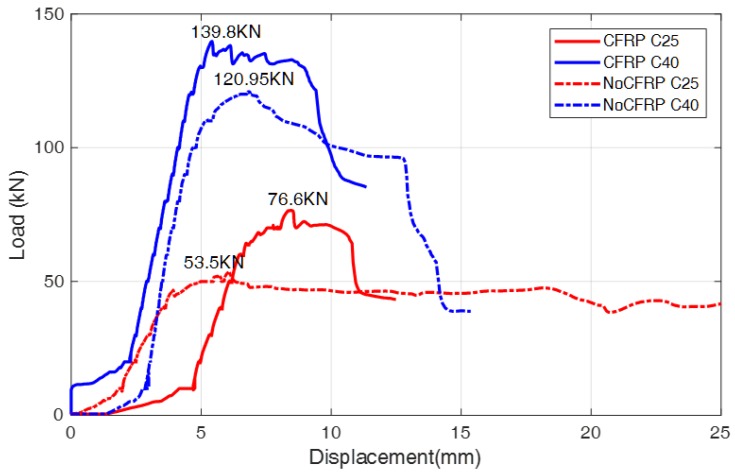
Load-displacement curves under different test conditions.

Based on above analysis, the strengthening performance of the SCFRP fabric for these four different concrete specimens were also compared and analyzed, as shown in Figure 22. From the figure, it is clear that the developed SCFRP fabric can significantly improve the ultimate bearing capacity of the beam specimen, no matter what kinds of concrete grades were used (C25 and C40). On the other hand, it is observed that the specimen with the lower concrete grade has a more significant improvement in the bearing capacity when compared to the specimen with the higher concrete grade. This phenomenon may be explained by their different failure modes. The debonding between the U-shaped CFRP fabric and the C25 beam limits the performance of the CFRP fabric in its high tensile/shear strength ability. The specimen with C40 concrete failed due to the fact that the carbon fiber reinforcement reached to its ultimate bearing capacity. Therefore, optimization of the layout and location of the SCFRP fabric is very necessary for the strengthening of concrete structures in practice. 

### 4.2. Discussion

In this research, a novel self-sensing CFRP technology was developed to take advantages of the PZT transducers’ active sensing function and the CRFP’s strengthening capacity. The feasibility of the proposed technology to realize the health condition monitoring of the concrete structures was studied. Experimental results of the three-points bending tests clearly show that the relationship between the wavelet packet energy value and the integrity of the test specimen. However, the wave propagation and attenuation may also be influenced by some other factors, including noise, thermal stress, moisture, external stress, and sensor location, which may affect the final identification results. At the current stage, the study is more focused on the feasibility of the proposed SCFRP fabric to monitor the health condition of the concrete specimen. Implementation of developed SCFRP fabric to monitor the working condition of in-service concrete structures remains a future topic to study. Another future topic will be modelling the interface between the CFRP fabric and the concrete under an external load by using the fractal contact theory with the help of PZT transducers [67,68]. In addition, the proposed SCFRP fabric in shear strengthening [69] and aseismic performance improvement [70] of concrete structures is important and will be investigated. 

## 5. Conclusions

In this paper, a novel Self-sensing Carbon Fiber Reinforced Polymer (SCFRP) fabric was developed to take advantages of piezoceramic transducers and the Carbon Fiber Reinforced Polymer (CFRP) strengthening materials in civil engineering. Thus, the developed SCFRP fabric has the ability to improve the structural bearing capability and simultaneously to realize the health condition monitoring of the strengthened structures. In order to validate the feasibility of the developed SCFRP concept, several specimens with or without the SCFRP fabric strengthening were fabricated and tested in the three-point bending configuration. In addition, the specimens with different concrete strength (C25 and C40) were also experimental investigated. A swept frequency test was applied for each specimen under different conditions and its corresponding stress wave response signals were collected and analyzed. At the same time, the wavelet packet energy technique was also employed to build a detection index to evaluate the changes of the received signals. Finally, the experimental results through comparison clearly show that the structural yield strength increases with the SCFRP fabric installation and the energy values of sensor signal decrease with crack development, which indicate that the developed novel SCFRP system has the ability to improve the bearing capability of strengthened concrete structures and to detect the occurrence and development of cracks in the structures. All these results demonstrate that the developed SCFRP fabric has a great potential in the structural strengthening with the self-sensing capacity for structural health monitoring for future civil engineering structures. 

## Figures and Tables

**Table 1:** The material properties of the test specimens and the CFRP.

Materials		Parameters	Value	Units
CFRP		Density	200	g/m^2^
		Theory thickness	0.111	mm/layer
		Tensile strength	3400	Mpa
		Young’s modulus	240	Gpa
		Elongation	1.7	%
		Inter-lamination shear strength	45	Mpa
Concrete	C25	Density	2381	kg/m^3^
		Young’s modulus	28	Gpa
		Compression strength	25.6	Mpa
	C40	Density	2400	kg/m^3^
		Young’s modulus	33	Gpa
		Compression strength	40.3	Mpa
Adhesive		Tensile strength	43	Mpa
		Young’s modulus (tensile)	2510	Mpa
		Elongation	2.13	%
		Compressive strength	82	Mpa
		Flexure strength	50.4	Mpa
		Bonding strength (with concrete)	3.6	Mpa
PZT-5H		Dimension	∅20 × 1	mm
		Density	7800	kg/m^3^
		Young’s modulus	46	Gpa
		Dielectric loss factor	0.02	--
		Mechanical loss factor	0.001	--
		Piezoelectric strain coefficients *d*_31_, *d*_32_/*d*_33_/*d*_24_, *d*_15_	−2.10/5.00/5.80	10^−10^ m/V or 10^−10^ C/N
		Electric permittivity $\varepsilon_{11}^{T}$, $\varepsilon_{22}^{T}$/$\varepsilon_{33}^{T}$	1.75/2.12	10^−8^ F/m

## References

[1] Teng J., Chen J.-F., Smith S.T., Lam L. (2002). FRP: Strengthened RC Structures.

[2] Zheng Y., Li C., Yang J., Sun C. (2015). Influence of arching action on shear behavior of laterally restrained concrete slabs reinforced with GFRP bars. Compos. Struct..

[3] Luo M., Li W., Hei C., Song G. (2016). Concrete infill monitoring in concrete-filled FRP tubes using a PZT-based ultrasonic time-of-flight method. Sensors.

[4] Xia L., Zheng Y. (2018). Deep Embedment (DE) FRP Shear Strengthening of Concrete Bridge Slabs under Loads Close to Supports. Appl. Sci..

[5] Miller T.C., Chajes M.J., Mertz D.R., Hastings J.N. (2001). Strengthening of a steel bridge girder using CFRP plates. J. Bridge Eng..

[6] Spadea G., Bencardino F., Swamy R. (1998). Structural behavior of composite RC beams with externally bonded CFRP. J. Compos. Constr..

[7] Khalifa A., Nanni A. (2000). Improving shear capacity of existing RC T-section beams using CFRP composites. Cem. Concr. Compos..

[8] Dransfield K., Baillie C., Mai Y.-W. (1994). Improving the delamination resistance of CFRP by stitching—A review. Compos. Sci. Technol..

[9] Howser R., Moslehy Y., Gu H., Dhonde H., Mo Y., Ayoub A., Song G. (2011). Smart-aggregate-based damage detection of fiber-reinforced-polymer-strengthened columns under reversed cyclic loading. Smart Mater. Struct..

[10] Colombi P., Poggi C. (2006). An experimental, analytical and numerical study of the static behavior of steel beams reinforced by pultruded CFRP strips. Compos. Part B Eng..

[11] Lou T., Karavasilis T.L. (2018). Time-dependent assessment and deflection prediction of prestressed concrete beams with unbonded CFRP tendons. Compos. Struct..

[12] Lou T., Lopes S.M., Lopes A.V. (2017). Effect of linear transformation on nonlinear behavior of continuous prestressed beams with external FRP cables. Eng. Struct..

[13] Al-Salloum Y.A., Al-Amri G.S., Siddiqui N.A., Almusallam T.H., Abbas H. (2018). Effectiveness of CFRP Strengthening in Improving Cyclic Compression Response of Slender RC Columns. J. Compos. Constr..

[14] Colombi P., Fava G. (2015). Experimental study on the fatigue behavior of cracked steel beams repaired with CFRP plates. Eng. Fract. Mech..

[15] Neubauer U., Rostasy F. In Design aspects of concrete structures strengthened with externally bonded CFRP-plates. Proceedings of the Seventh International Conference on Structural Faults and Repair: Concrete and Composites.

[16] El-Ghandour A. (2011). Experimental and analytical investigation of CFRP flexural and shear strengthening efficiencies of RC beams. Constr. Build. Mater..

[17] Gherdaoui M., Guenfoud M., Madi R. (2018). Punching behavior of strengthened and repaired RC slabs with CFRP. Constr. Build. Mater..

[18] Dai J., Ueda T., Sato Y. (2005). Development of the nonlinear bond stress–slip model of fiber reinforced plastics sheet–concrete interfaces with a simple method. J. Compos. Constr..

[19] Al-Rousan R., Issa M. (2011). Fatigue performance of reinforced concrete beams strengthened with CFRP sheets. Constr. Build. Mater..

[20] Borri A., Corradi M., Grazini A. (2005). A method for flexural reinforcement of old wood beams with CFRP materials. Compos. Part B Eng..

[21] Perera R., Sun R., Sevillano E., Ruiz A. (2017). A multi-objective electromechanical impedance technique to identify debonding in RC beams flexural strengthened with FRP. Procedia Eng..

[22] Kim S.B., Sohn H. (2007). Instantaneous reference-free crack detection based on polarization characteristics of piezoelectric materials. Smart Mater. Struct..

[23] Song G., Wang C., Wang B. (2017). Structural health monitoring (SHM) of civil structures. Appl. Sci..

[24] Balageas D., Fritzen C.-P., Güemes A. (2010). Structural Health Monitoring.

[25] Li H., Ou J. (2016). The state of the art in structural health monitoring of cable-stayed bridges. J. Civ. Struct. Health Monit..

[26] Mitra M., Gopalakrishnan S. (2016). Guided wave based structural health monitoring: A review. Smart Mater. Struct..

[27] Li W., Ho S.C.M., Luo M., Huynh Q., Song G. (2017). Fiber optic macro-bend based sensor for detection of metal loss. Smart Mater. Struct..

[28] Luo M., Li W., Wang B., Fu Q., Song G. (2017). Measurement of the Length of Installed Rock Bolt Based on Stress Wave Reflection by Using a Giant Magnetostrictive (GMS) Actuator and a PZT Sensor. Sensors.

[29] Bao Y., Yu Y., Li H., Mao X., Jiao W., Zou Z., Ou J. (2015). Compressive sensing-based lost data recovery of fast-moving wireless sensing for structural health monitoring. Struct. Control Health Monit..

[30] Nagayama T., Spencer B., Agha G., Mechitov K. In Model-based data aggregation for structural monitoring employing smart sensors. Proceedings of the of INSS.

[31] Cho S., Yun C.-B., Lynch J.P., Zimmerman A.T., Spencer B.F., Nagayama T. (2008). Smart wireless sensor technology for structural health monitoring of civil structures. Steel Struct..

[32] Yi T.H., Li H.N., Song G., Zhang X.D. (2015). Optimal sensor placement for health monitoring of high-rise structure using adaptive monkey algorithm. Struct. Control Health Monit..

[33] Srbinovski B., Magno M., Edwards-Murphy F., Pakrashi V., Popovici E. (2016). An energy aware adaptive sampling algorithm for energy harvesting WSN with energy hungry sensors. Sensors.

[34] Zinno R., Artese S., Clausi G., Magarò F., Meduri S., Miceli A., Venneri A. (2019). Structural Health Monitoring (SHM). The Internet of Things for Smart Urban Ecosystems.

[35] Zhang L., Wang C., Song G. (2015). Health status monitoring of cuplock scaffold joint connection based on wavelet packet analysis. Shock Vib..

[36] Hackmann G., Guo W., Yan G., Sun Z., Lu C., Dyke S. (2014). Cyber-physical codesign of distributed structural health monitoring with wireless sensor networks. IEEE Trans. Parallel Distrib. Syst..

[37] Wang H., Tao T., Li A., Zhang Y. (2016). Structural health monitoring system for Sutong cable-stayed bridge. Smart Struct. Syst..

[38] Huo L., Chen D., Liang Y., Li H., Feng X., Song G. (2017). Impedance based bolt pre-load monitoring using piezoceramic smart washer. Smart Mater. Struct..

[39] Ji Q., Ho M., Zheng R., Ding Z., Song G. (2015). An exploratory study of stress wave communication in concrete structures. Smart Mater. Struct..

[40] Li W., Fan S., Ho S.C.M., Wu J., Song G. (2018). Interfacial debonding detection in fiber-reinforced polymer rebar–reinforced concrete using electro-mechanical impedance technique. Struct. Health Monit..

[41] Liang Y., Li D., Parvasi S.M., Kong Q., Song G. (2016). Bond-slip detection of concrete-encased composite structure using electro-mechanical impedance technique. Smart Mater. Struct..

[42] Liang Y., Li D., Parvasi S.M., Song G. (2016). Load monitoring of pin-connected structures using piezoelectric impedance measurement. Smart Mater. Struct..

[43] Hou S., Zhang H., Ou J. (2016). SA-based concrete seismic stress monitoring: A case study for normal strength concrete. Smart Mater. Struct..

[44] Song G., Gu H., Mo Y., Hsu T., Dhonde H. (2007). Concrete structural health monitoring using embedded piezoceramic transducers. Smart Mater. Struct..

[45] Yang Y., Divsholi B.S., Soh C.K. (2010). A reusable PZT transducer for monitoring initial hydration and structural health of concrete. Sensors.

[46] Park G., Cudney H.H., Inman D.J. (2000). Impedance-based health monitoring of civil structural components. J. Infrastruct. Syst..

[47] Wang T., Song G., Wang Z., Li Y. (2013). Proof-of-concept study of monitoring bolt connection status using a piezoelectric based active sensing method. Smart Mater. Struct..

[48] Feng Q., Kong Q., Huo L., Song G. (2015). Crack detection and leakage monitoring on reinforced concrete pipe. Smart Mater. Struct..

[49] Feng Q., Kong Q., Song G. (2016). Damage detection of concrete piles subject to typical damage types based on stress wave measurement using embedded smart aggregates transducers. Measurement.

[50] Dziendzikowski M., Kurnyta A., Dragan K., Klysz S., Leski A. (2016). In situ Barely Visible Impact Damage detection and localization for composite structures using surface mounted and embedded PZT transducers: A comparative study. Mech. Syst. Signal Process..

[51] Tsangouri E., Karaiskos G., Aggelis D.G., Deraemaeker A., Van Hemelrijck D. (2015). Crack sealing and damage recovery monitoring of a concrete healing system using embedded piezoelectric transducers. Struct. Health Monit.

[52] Zhang H., Hou S., Ou J. (2017). Feasibility of SA–based concrete seismic stress monitoring for high-strength concrete. J. Intell. Mater. Syst. Struct..

[53] Hou S., Kong Z., Wu B., Liu L. (2018). Compactness Monitoring of Compound Concrete Filled with Demolished Concrete Lumps Using PZT-Based Smart Aggregates. J. Aerosp. Eng..

[54] Narayanan A., Kocherla A., Subramaniam K.V. (2018). Understanding the coupled electromechanical response of a PZT patch attached to concrete: Influence of substrate size. Measurement.

[55] Lu G., Li Y., Wang T., Xiao H., Huo L., Song G. (2017). A multi-delay-and-sum imaging algorithm for damage detection using piezoceramic transducers. J. Intell. Mater. Syst. Struct..

[56] Lim Y.Y., Liew W.Y.H., Soh C.K. (2015). A parametric study on admittance signatures of a PZT transducer under free vibration. Mech. Adv. Mater. Struct..

[57] Shao J., Wang T., Yin H., Yang D., Li Y. (2016). Bolt looseness detection based on piezoelectric impedance frequency shift. Appl. Sci..

[58] Yan S., Ma H., Li P., Song G., Wu J. (2017). Development and application of a structural health monitoring system based on wireless smart aggregates. Sensors.

[59] Kong Q., Fan S., Mo Y., Song G. (2017). A novel embeddable spherical smart aggregate for structural health monitoring: Part II. Numerical and experimental verifications. Smart Mater. Struct..

[60] Zeng L., Parvasi S.M., Kong Q., Huo L., Li M., Song G. (2015). Bond slip detection of concrete-encased composite structure using shear wave based active sensing approach. Smart Mater. Struct..

[61] Asgarian B., Aghaeidoost V., Shokrgozar H.R. (2016). Damage detection of jacket type offshore platforms using rate of signal energy using wavelet packet transform. Marine Struct..

[62] Lei D., Yang L., Xu W., Zhang P., Huang Z. (2017). Experimental study on alarming of concrete micro-crack initiation based on wavelet packet analysis. Constr. Build. Mater..

[63] Naderpour H., Fakharian P. (2016). A synthesis of peak picking method and wavelet packet transform for structural modal identification. KSCE J. Civ. Eng..

[64] Chen J., Li Z., Pan J., Chen G., Zi Y., Yuan J., Chen B., He Z. (2016). Wavelet transform based on inner product in fault diagnosis of rotating machinery: A review. Mech. Syst. Signal Process..

[65] Xu B., Zhang T., Song G., Gu H. (2013). Active interface debonding detection of a concrete-filled steel tube with piezoelectric technologies using wavelet packet analysis. Mech. Syst. Signal Process..

[66] Li D., Liang Y., Feng Q., Song G. (2018). Load monitoring of the pin-connected structure based on wavelet packet analysis using piezoceramic transducers. Measurement.

[67] Wang F., Huo L., Song G. (2017). A piezoelectric active sensing method for quantitative monitoring of bolt loosening using energy dissipation caused by tangential damping based on the fractal contact theory. Smart Mater. Struct..

[68] Wang F., Ho S.C.M., Huo L., Song G. (2018). A Novel Fractal Contact-Electromechanical Impedance Model for Quantitative Monitoring of Bolted Joint Looseness. IEEE Access.

[69] Del Vecchio C., Del Zoppo M., Di Ludovico M., Verderame G.M., Prota A. (2017). Comparison of available shear strength models for non-conforming reinforced concrete columns. Eng. Struct..

[70] Del Vecchio C., Di Ludovico M., Balsamo A., Prota A. (2018). Seismic retrofit of real beam-column joints using fiber-reinforced cement composites. J. Struct. Eng..

